# Supplementary material for: Herbicide leakage into seawater impacts primary productivity and zooplankton globally
Source: Nat Commun. 2024 Feb 27;15:1783. doi: 10.1038/s41467-024-46059-4 (PMC10899588; doi:10.1038/s41467-024-46059-4)
Supplement: Supplementary file 1 — Supplementary Information [file 41467_2024_46059_MOESM1_ESM.pdf]

Supplementary Information for

Herbicide leakage into seawater impacts primary productivity and zooplankton globally

Liqiang Yang[1,2], Xiaotong He[1], Shaoguo Ru[1*], Yongyu Zhang[2,3,4*]

[1]College of Marine Life Sciences, Ocean University of China, Qingdao, China

[2]Qingdao Institute of Bioenergy and Bioprocess Technology, Chinese Academy of Sciences, No. 189 Songling Road, 266101, Qingdao, Shandong, China

[3]Shandong Energy Institute, No. 189 Songling Road, 266101, Qingdao, Shandong, China

[4]Qingdao New Energy Shandong Laboratory, 266101, Qingdao, Shandong, China

[*] e-mail: zhangyy@qibebt.ac.cn

This document contain:

Table S1-S7

Figure. S1-S10

Table S1. The peak and median concentrations of the twelve triazine herbicides and their derivatives in global bays between 1990 and 2022.

Sea areas	Bay name	Longitude	Latitude	Median total concentration (nmol L^{-1})	Maximum concentration (nmol L^{-1})
East Coast of USA	Winyah Bay	-79.2400	33.2700	1.32	3.36
	Chesapeake Bay	-76.3166	37.1765	0.38	7.55
Gulf of Mexico	Biscayne Bay	80.2286	25.7261	0.14	0.37
	The Gulf of Mexico	-91.5138	25.4018	2.65	13.67
France	Vilaine Bay	-2.3890	47.9300	0.21	11.68
Mediterranean Sea	Napoli Gulf	14.2490	40.8184	0.055	0.68
	Amvrakikos Gulf	20.9556	38.9881	1.02	3.3
	Thermaikos Gulf	22.9333	40.6333	7.5	29.5
South Africa	Camps Bay	18.3751	-33.9522	0.015	0.027
	Maputo Bay	32.5276	-25.9608	0.056	13
East Asia	Xiangshan Harbor	121.7200	29.5400	0.14	0.32
	Jiaozhou Bay	120.0938	36.2431	0.28	1.52
	North Yellow Sea and Bohai Sea	121.0105	36.0012	2.31	12.07
Australia	Hervey Bay	-25.2833	152.8478	0.071	0.55
	Great Barrier Reef	-15.9390	146.5005	0.096	1.711

Table S2. The concentration-response functions for each single triazine herbicide.

Sensitive species level	EC50	Model	*A1*	*A2*	*X0*/d	*p*/*k*	R^2adj
Atrazine	141.3 ± 12.5	L	1.7142	104.2656	0.1349	0.9173	0.989
DEA	580.3 ± 21.8	L	1.5695	101.0427	0.6049	1.1381	0.995
Propazine	147.1 ± 15.2	L	4.3068	103.3116	0.1584	1.1995	0.989
Simazine	28.7 ± 1.6	L	0.3833	103.3089	0.0309	0.8911	0.996
Terbutryn	8.5 ± 1.1	L	2.1441	101.7772	0.0091	1.1025	0.997
Ametryn	25.1 ± 1.2	L	−0.5755	102.6998	0.0262	0.9461	0.996
Dipropetryn	37.0 ± 3.1	L	−1.0603	101.592	0.0375	0.8534	0.993
Cyanazine	280.3± 11.7	L	1.1414	109.9918	0.36235	0.66913	0.995
Cybutryne	4.3± 0.3	L	0.678	100.21925	0.00445	1.08514	0.999
DIA	732.1 ± 36.5	W	4.0148	99.4471	0.8182	0.8223	0.989
Prometryn	849.1 ± 21.7	W	1.9596	99.3406	1.0341	0.8109	0.997

Prometon	76.2 ± 3.3	W	5.80E-46	96.4563	0.643	0.2094	0.996
Atrazine (Community level)	55.8±3.25	L	3.1467	101.122	0.103	1.0778	0.995

Note: n = 3 samples per group, and data are presented as mean ± SD. Differences were considered significant at *p* < 0.05*, *p* < 0.01, and *p* < 0.001*** according to Kruskal–Wallis tests.**

EC50: 50% effective concentration, the unit is nmol L^{-1}; L: Logistic; W: Weibull; A1, A2, X0, p, d and k represent the parameters of the functions.

Logistic

$$y = \frac{A_1 - A2}{1 + (x/X_0)^p} + A_2$$

Weibull

$$y = A_2 - (A_2 - A_1)e^{-(kx)^d}$$

Table S3. Alpha diversity of the phyto- and microzooplankton communities in the control and atrazine-treated groups on the 21st day.

Phytoplankton	Shannon	Chao	Ace	zooplankton	Shannon	Chao	Ace
CK	1.33±0.08	80.64±13.4	78.09±8.04	CK	1.88±0.11	160.65±9.66	159.23±2.65
0.5 nmol L^{-1}	2.02±0.36* ($p = 0.041$)	62.72±8.84* ($p = 0.021$)	59.02±10.32* ($p = 0.0147$)	0.5 nmol L^{-1}	1.55±0.41 ($p = 0.20$)	117.71±27.13* ($p = 0.047$)	114.07±26.47* ($p = 0.042$)
5 nmol L^{-1}	2.44±0.47* ($p = 0.039$)	48.75±4.56** ($p = 0.025$)	56.40±11.71* ($p = 0.0194$)	5 nmol L^{-1}	1.30±0.46* ($p = 0.035$)	87.80±18.25* ($p = 0.046$)	89.47±27.36* ($p = 0.039$)
50 nmol L^{-1}	3.03±0.21*** ($p = 0.00091$)	39.52±5.69*** ($p = 0.00069$)	42.02±7.51** ($p = 0.0038$)	50 nmol L^{-1}	1.42±0.16** ($p = 0.0039$)	96.28±5.60*** ($p = 0.00013$)	109.10±9.47*** ($p = 0.00061$)

Values are the mean of triplicate samples plus or minus the standard deviation.

Note: n = 3 samples per group, and data are presented as mean ± SD. Differences were considered significant at $p < 0.05$*, $p < 0.01$, and $p < 0.001$*** according to t-tests (two-sided) with FDR adjusted for multiple comparisons using the Benjamini and Hochberg method.**

Table S4. Taxonomic assignment of highly abundant genera of phytoplankton and zooplankton in control and atrazine-treated groups.

OUT ID	Control	0.5 nmol L^{-1}	5 nmol L^{-1}	50 nmol L^{-1}	Phylum	Class	Family	Genus	Similar species	NCBI No. (Similarity)	Cell size (μm)	Reference
OTU187	9.8	17.1*** (p= 0.00075)	14.3*** (p= 0.00046)	60.5*** (p= 0.00028)	Dinophyceae	Dinophyceae	Gymnodiniaceae	*Gyrodinium*	*Gyrodinium jinhaense*	MH665395.1 (100%)	Micro- (21.8-39.9)	Jiang *et al*, 2019[1]
OTU342	< 1.0	3.9* (p= 0.039)	1.3 (p= 0.085)	< 1.0 (p= 0.081)	Dinophyceae	Dinophyceae	Gonyaulacaceae	*Adenoides*	*Adenoides eludens*	KY980212.1 (100%)	Micro- (28-35)	Hoppenrath et al[2], 2003
OTU386	< 1.0	4.7* (p= 0.021)	3.0* (p= 0.033)	2.1* (p= 0.019)	Dinophyceae	Dinophyceae	Eudubosquellidae	*Euduboscquella*	*Euduboscquella sp. JMC-2019a*	MN388923.1 (88.7%)	Micro- (85-194)	Jung et al, 2016[3]
OTU755	1.2	1.5 (p= 0.48)	1.6 (p= 0.25)	2.7** (p= 0.0039)	Cryptophyceae	Cryptophyceae	Goniomonadaceae	*Goniomonas*	*Goniomonas avonlea*	JQ434475.1 (96.4%)	Nano- (7-11)	Kim et al, 2013[4]
OTU655	< 1.0	< 1.0 (p= 0.57)	< 1.0 (p= 0.18)	2.8* (p= 0.015)	Dinophyceae	Dinophyceae	Lophodiniaceae	*Woloszynskia*	*Woloszynskia halophila*	AY628430.1 (87.5%)	Micro- (32-35)	Siano et al, 2009[5]
OTU279	< 1.0	< 1.0 (p= 0.36)	< 1.0 (p= 0.17)	1.6* (p= 0.029)	Dinophyceae	Dinophyceae	Kareniaceae	*Karlodinium*	*Karlodinium veneficum*	JF791048.1 (96.9%)	Nano- (14-18)	Place et al, 2012[6]
OTU781	< 1.0	< 1.0 (p= 0.059)	1.2 (p= 0.115)	1.5* (p= 0.025)	Dinophyceae	Dinophyceae	Heterocapsaceae	*Heterocapsa*	*Heterocapsa rotundata*	KY980409.1 (97.6%)	Nano- (10-14)	Hansen et al, 1995[7]
OTU285	< 1.0	16.1*** (p= 0.00013)	4.4* (p= 0.031)	2.9* (p= 0.026)	Haptista	Prymnesiophyceae	Chrysochromulinaceae	*Chrysochromulina*	*Chrysochromulina rotalis*	LT560338.1 (100%)	Pico- (2-6)	Eikrem et al, 1999[8]
OTU319	< 1.0	11.2** (p= 0.0047)	2.8* (p= 0.019)	1.6* (p= 0.0398)	Haptista	Prymnesiophyceae	Chrysochromulinaceae	*Chrysochromulina*	*Chrysochromulina leadbeateri*	AM491017.2 (99.4%)	Pico- (2-8)	Estep et al, 1984[9]
OTU17	< 1.0	1.2* (p= 0.0272)	13.3*** (p= 0.00004)	4.5*** (p= 0.00055)	Raphidophyceae	Raphidophyceae	Chattonellaceae	*Fibrocapsa*	*Fibrocapsa japonica*	JX026949.1 (100%)	Micro- (32-48)	Rhodes et al, 1999[10]

OTU533	< 1.0	6.3* (*p*= 0.0168)	1.8 (*p*= 0.081	< 1.0 (*p*= 0.345)	Dinophyceae	Dinophyceae	Gymnodiniaceae	*Ankistrodinium*	*Ankistrodinium semilunatum*	AF274256.1 (91.5%)	Micro- (40-60)	Hoppenrath et al, 2021[2]
OTU355	< 1.0	2.7 (*p*= 0.773)	17.0*** (*p*= 0.00027)	8.1** (*p*= 0.0038)	Dinophyceae	Dinophyceae	Kareniaceae	*Gertia*	*Gertia stigmatica*	LC490696.1 (97.4%)	Nano- (7.8-9.5)	Takahashi et al, 2019[11]
OTU484	< 1.0	1.1* (*p*= 0.015)	13.0*** (*p*= 0.00045)	4.7** (*p*= 0.0042)	Pelagophyceae	Pelagophyceae	Aureococcus	*Aureococcus*	*Aureococcus anophagefferens*	KY980308.1 (95.2%)	Pico- (2.0-3.0)	Ma *et al*, 2020[12]
OTU334	0.73	5.2*** (*p*= 0.0007)	2.9*** (*p*= 0.00029)	< 1.0*** (*p*= 0.00013)	Bacillariophyta	Coscinodiscophyceae	Chaetocerotaceae	*Chaetoceros*	*Chaetoceros tenuissimus*	MG972315.1 (100%)	Nano- (>4)	[13]Meunier *et al*, 1913
OTU565	2.7	2.1 (*p*= 0.553)	8.5* (*p*= 0.042)	< 1.0 (*p*= 0.77)	Bacillariophyta	Coscinodiscophyceae	Thalassiosiraceae	*Thalassiosira*	*Thalassiosira nordenskioeldii*	MW722947.1 (100%)	Nano- (13.2-44.6)	Durbin et al, 1978[14]
OTU634	< 1.0	1.3 (*p*= 0.28)	4.3* (*p*= 0.011)	4.7* (*p*= 0.0192)	Ciliophora	Spirotrichea	Euplotidae	*Moneuplotes*	*Moneuplotes minuta*	KX516699.1 (99.1%)	Micro- (40-70)	Song et al, 1997[15]
OTU630	< 1.0	< 1.0 (*p*= 0.46)	5.4** (*p*= 0.0041)	46.1*** (*p*= 0.00058)	Ciliophora	Spirotrichea	Holostichidae	*Holosticha*	*Holosticha diademata*	KF306396.1 (100%)	Micro- (28-90)	Hu et al, 2001[16]
OTU270	1.3	7.5** (*p*= 0.0033)	12.0*** (*p*= 0.00017)	3.1* (*p*= 0.047)	Annelida	Polychaeta	Spionidae	*Pseudopolydora*	*Pseudopolydora paucibranchiata*	LC019991.1 (100%)	larvae	Blake et al, 1975[17]
OTU13	8.4	2.7*** (*p*= 0.00044)	< 1.0*** (*p*= 0.00029)	< 1.0*** (*p*= 0.00033)	Annelida	Polychaeta	Chrysopetalidae	*Paleanotus*	*Paleanotus bellis*	EU555041.1 (99.58)	larvae	Milejkovskij et al, 1961[18]
OTU282	< 1.0	5.1*** (*p*= 0.00019)	< 1.0 (*p*= 0.28)	< 1.0 (*p*= 0.49)	Arthropoda	Hexanauplia	Acartiidae	*Acartia*	*Acartia pacifica*	GU969157.1 (100%)	larvae	Moon et al, 2008[19]
OTU264	74.1	72.3 (*p*= 0.093)	73.0 (*p*= 0.088)	14.3*** (*p*= 0.00028)	Arthropoda	Hexanauplia	Oithonidae	*Oithona*	*Oithona davisae*	KT030258.1 (100%)	larvae	Ferrari et al, 1984[20]
OTU231	< 1.0	5.0* (*p*= 0.012)	< 1.0 (*p*= 0.34)	< 1.0 (*p*= 0.65)	Arthropoda	Hexanauplia	Pyrgomatidae	*Pyrgoma*	*Pyrgoma cancellatum*	KM217494.1 (99.8%)	larvae	Ross et al, 2002[21]

OTU51	4.7	3.2* (p= 0.041)	< 1.0** (p= 0.0037)	< 1.0** (p= 0.0031)	Mollusca	Bivalvia	Ostreidae	*Crassostrea*	*Crassostrea gigas*	CP048848.1 (100%)	larvae	Escapa et al, 2004[22]
OTU97	2.0	< 1.0* (p= 0.047)	< 1.0* (p= 0.025)	< 1.0* (p= 0.0393)	Mollusca	Bivalvia	Veneridae	*Ruditapes*	*Ruditapes philippinarum*	MZ227551.1 (100%)	larvae	Delgado et al, 2007[23]
OTU611	< 1.0	< 1.0 (p= 0.73)	< 1.0 (p= 0.22)	14.5*** (p= 0.00034)	Platyhelminthes	Rhabditophora	Plehniidae	*Paraplehnia*	*Paraplehnia seisuiae*	LC508167.1 (99.6%)	larvae	Oya et al, 2019[24]

Note: The mean relative abundance (%) for the respective group of samples is shown. n = 3 samples per group. Differences between the control and each atrazine-treated group were considered significant at $p < 0.05$*, $p < 0.01$**, and $p < 0.001$*** according to t-tests (two-sided) with FDR adjusted for multiple comparisons using the Benjamini and Hochberg method.

Darker colours indicate higher abundances of the respective features.

Table S5. Effects of atrazine exposure on phytoplankton growth rate and feeding pressure of zooplankton

Group	Partical Size (μm)	Intrinsic Growth Rate (*μ*)	Grazing Mortality Rate (*g*)	Net Growth Rate (NGR)	R^2
CK	20-200	0.65	0.61	0.04	0.87
	2-20	0.74	0.71	0.03	0.91
	＜2	1.14	1.02	0.12	0.81
0.5 nmol L^{-1}	20-200	0.67<br>(p = 0.89)	0.62<br>(p = 0.47)	0.05<br>(p = 0.51)	0.93
	2-20	0.61*<br>(p = 0.037)	0.69<br>(p = 0.79)	-0.08***<br>(p = 0.00011)	0.85
	＜2	1.01*<br>(p = 0.029)	0.94<br>(p = 0.92)	0.07**<br>(p = 0.0019)	0.88
5 nmol L^{-1}	20-200	0.53*<br>(p = 0.014)	0.5**<br>(p = 0.0026)	0.03<br>0.073	0.92
	2-20	0.47***<br>(p = 0.00081)	0.59**<br>(p = 0.0019)	-0.12***<br>(p = 0.00028)	0.87
	＜2	0.97*<br>(p = 0.029)	0.99<br>(p = 0.092)	-0.02***<br>(p = 0.00051)	0.84
50 nmol L^{-1}	20-200	0.44**<br>(p = 0.0072)	0.38***<br>(p = 0.00041)	0.06*<br>(p = 0.047)	0.88
	2-20	0.35***<br>(p = 0.00047)	0.52***<br>(p = 0.00027)	-0.17***<br>(p = 0.00092)	0.92

	＜2	0.82** ($p = 0.0028$)	0.77** ($p = 0.0044$)	-0.05*** ($p = 0.00028$)	0.85

Note: n = 3 samples per group, and the mean value is shown. Differences between the control and each atrazine-treated group were considered significant at $p < 0.05$*, $p < 0.01$, and $p < 0.001$*** according to t-tests (two-sided) with FDR adjusted for multiple comparisons using the Benjamini and Hochberg method.**

Table S6. The community structure analysis of microzooplankton based on morphological identification

Microzooplankton species	CK	0.5 nmol L^{-1}	5 nmol L^{-1}	50 nmol L^{-1}
Ciliophora				
Order: Choreotrichida				
Pelagostrobilidium epacrum	+	+	+	+
Pelagostrobilidium sp.	++	++	+	-
Strombidium capitatum	+	+	-	-
Strombidium parastylifer	+	+	-	-
Strombidium suzukii	++	++	+	-
Strombidium sp.	+++	+	+	-
Order: Tintinnida				
Tintinnopsis beroidea	+	+	-	-
Tintinnopsis tsingtaoensis	-	+	-	-

Tintinnopsis rapa	-	+	+	++
Favella sp.	-	+	++	-
Codonellopsis mobilis	-	-	+	-
Eutintinnus apertus	-	-	+	+
Order: Euplotida				
Euplotes eurystomus	+	+	+++	+++
Euplotes charon	+	+	++	++
Euplotes sp.	++	++	+++	+++
Order: Urostylida				
Holosticha diademata	-	+	++	++
Holosticha sp.	+	++	+++	+++
Copepod nauplii	++++	++++	+++	++

Abundances (ind. mL^{-1}): - = 0; + = 1-10; ++ = 10-50; +++ = 50-100; ++++ = over 100.

Table S7. The peak and median concentrations of the 22 herbicide residues in 3 categories in global bays between 1990 and 2022.

Sea areas	Bay name	Longitude	Latitude	Median total concentration (nmol L^{-1})	Maximum concentration (nmol L^{-1})
East Coast of USA	Winyah Bay	-79.2400	33.2700	1.56	3.36
	Chesapeake Bay	-76.3166	37.1765	0.99	41.00
Gulf of Mexico	Biscayne Bay	80.2286	25.7261	0.36	1.04
	The Gulf of Mexico	-91.5138	25.4018	2.85	13.67
France	Vilaine Bay	-2.3890	47.9300	0.49	12.99
Mediterranean Sea	Napoli Gulf	14.2490	40.8184	0.53	6.75
	Amvrakikos Gulf	20.9556	38.9881	0.96	3.3
	Thermaikos Gulf	22.9333	40.6333	7.5	29.5
South Africa	Camps Bay	18.3751	-33.9522	0.0183	0.0539
	Maputo Bay	32.5276	-25.9608	0.056	13
East Asia	Xiangshan Harbor	121.7200	29.5400	0.14	0.32
	Jiaozhou Bay	120.0938	36.2431	0.28	1.52
	North Yellow Sea and Bohai Sea	121.0105	36.0012	5.55	18.49
Australia	Hervey Bay	-25.2833	152.8478	0.04	0.55
	Great Barrier Reef	-15.9390	146.5005	0.115	1.711

Figure. S1

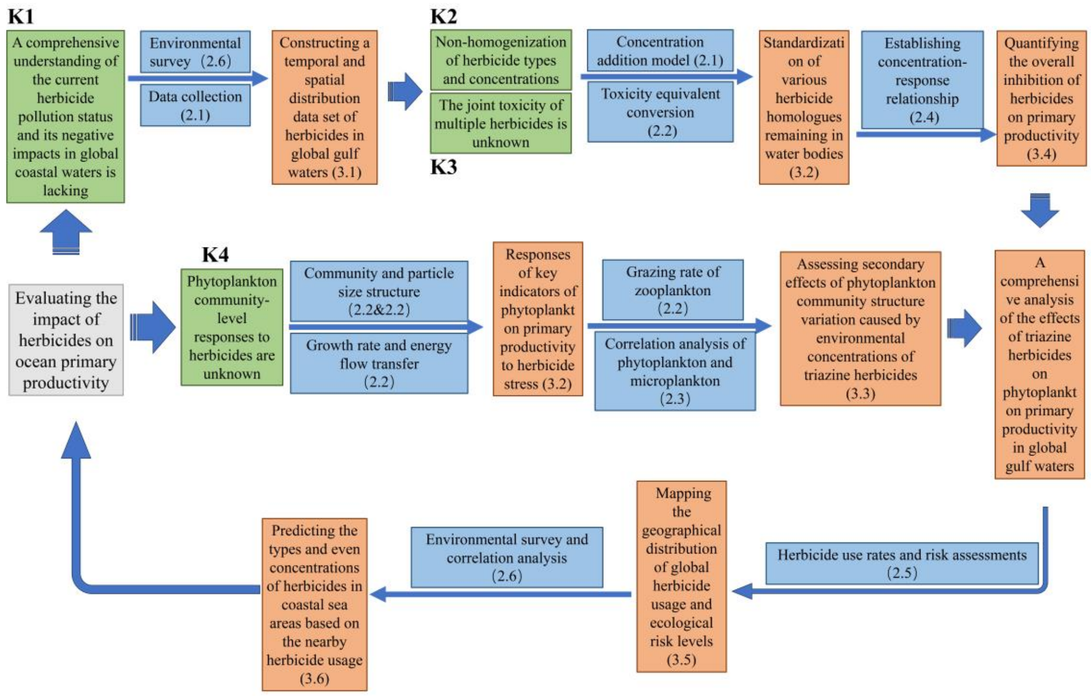

Figure. S1. A mind map showing the logical relationship among the article's aim, methods and results. Silver, green, blue and orange boxes represent the main purpose of the article, challenges faced, problem solving methods and phased results respectively. The number in each box corresponds to the Experimental Methods and Experimental Results sections (in the order of the titles) in the manuscript.

K1-K4 corresponds to the four knowledge gaps faced in this study.

Knowledge gap 1 (K1): Current herbicide toxicity studies are mostly based on risk assessment of a single herbicide in a small region, lacking a comprehensive understanding of the current status and impact of herbicide pollution in global coastal waters.

Knowledge gap 2 (K2): Due to the large differences in the types and concentrations of herbicides between different sites (Table S1), there is no unified prediction scale and comparison benchmark for the effects of herbicides on primary productivity.

Knowledge gap 3 (K3): There are joint toxicity effects between herbicides with the

same target of action. Thus, assessing the ecotoxicity of individual herbicides does not reflect the combined toxic effects of herbicides under in situ conditions.

Knowledge gap 4 (K4): It is difficult to truly understand the ecological effects of herbicides *in situ* only from the responses of individual algae or groups, and the impact of herbicides on the structure of the phytoplankton community cannot be based on changes in photosynthetic physiological indicators alone.

Figure. S2

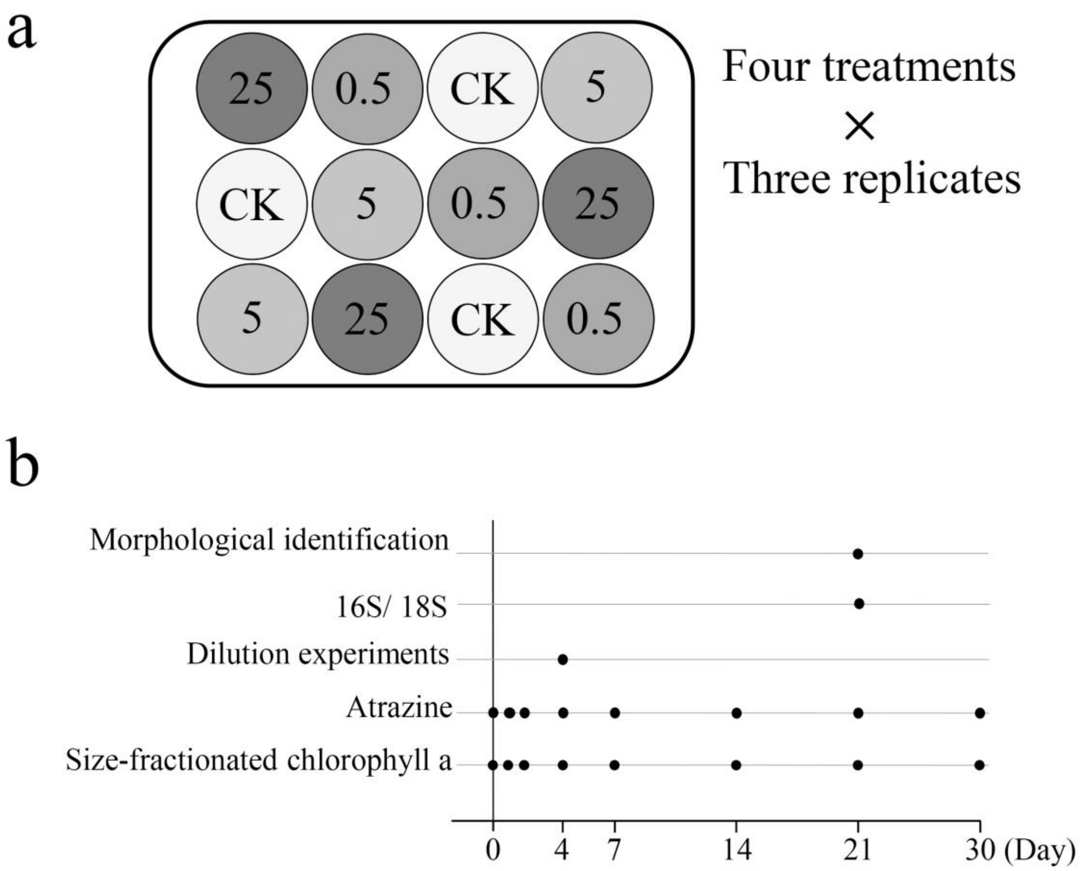

Figure. S2. Schematic diagram of the arrangement of 12 bottles (a) and the sampling time corresponding to each indicator (b)

Figure. S3

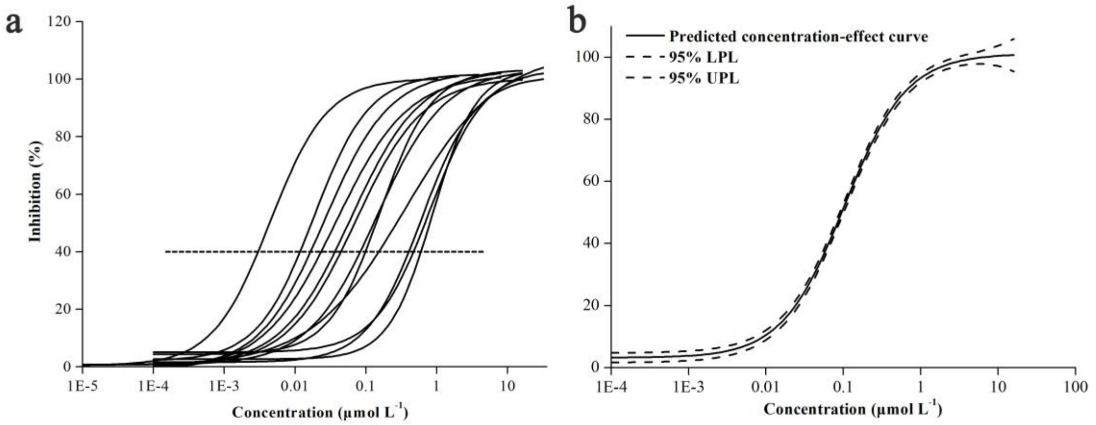

Figure. S3. Single-substance concentration–response curves of the 12 tested triazine herbicides (a) and the concentration–response curve of atrazine on the phytoplankton chlorophyll a concentration at the community level (b). From left to right, the curves intersecting the dashed lines represent the twelve triazine herbicides in the following order: Simazine, Dipropetryn, Cyanazine, Terbutryn, Cybutryne, Prometon, Prometryn, Desethylatrazine, Desisopropylatrazine, Propazine, Ametryn, and Atrazine; 95% LPL: 95% lower prediction limit; 95% UPL: 95% upper prediction limit.

Figure. S4

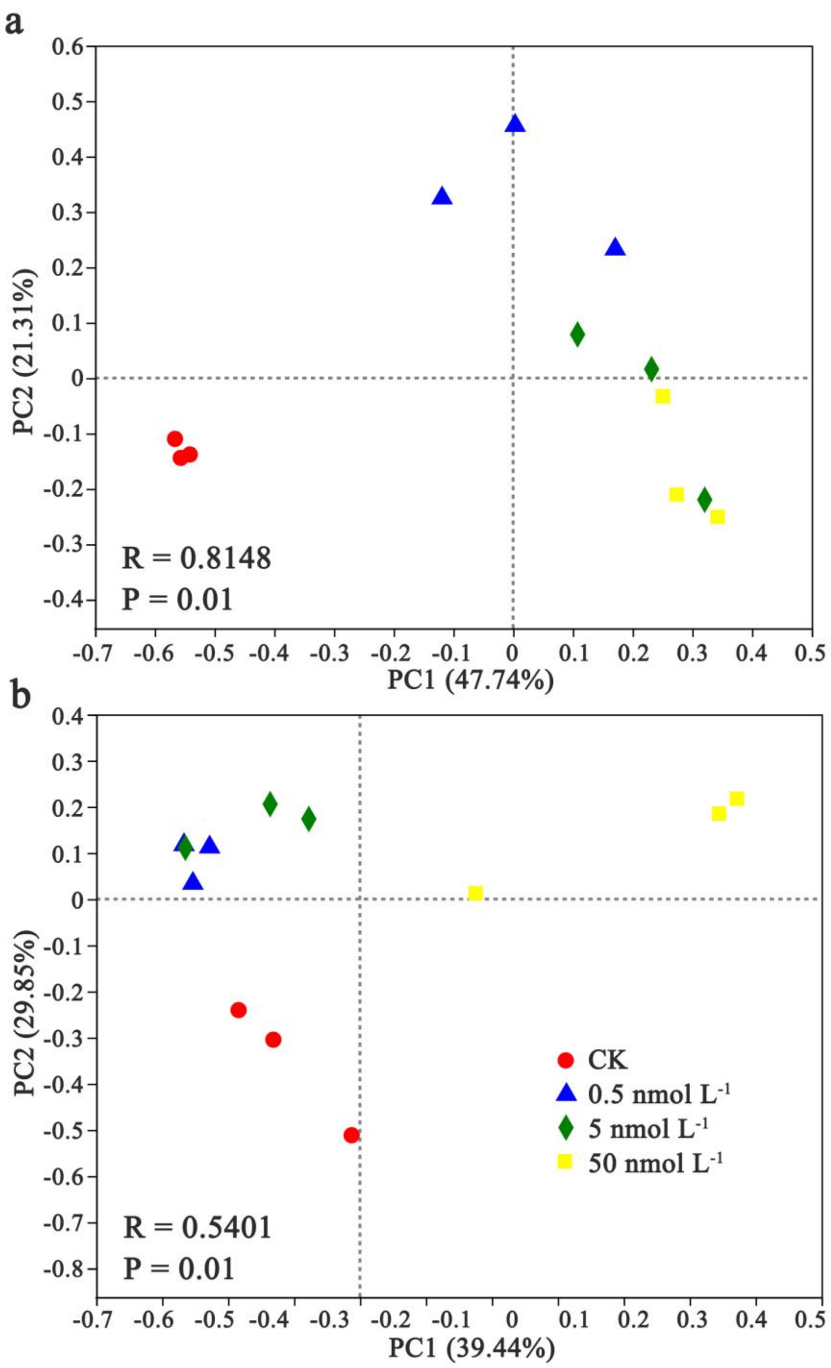

Figure. S4. PCoA of the phytoplankton (a) and zooplankton communities in the control and atrazine-treated groups (b). CK, 0.5, 5 and 50 nmol L^{-1} represent the control and treatment groups dosed with 0.5-50 nmol L^{-1} of atrazine, respectively, on the 21st day (with three replicates).

Figure. S5

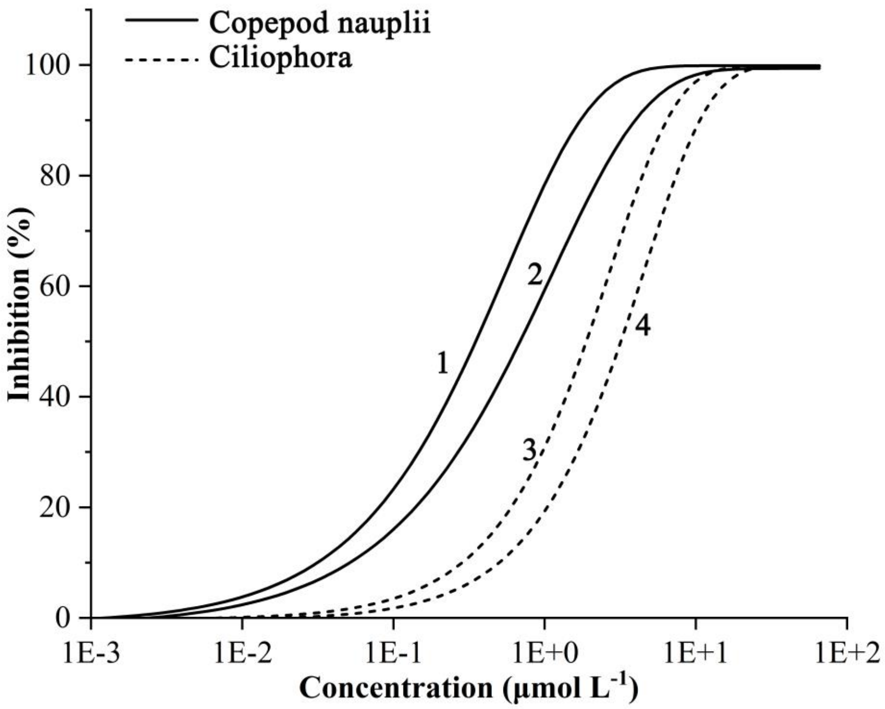

Figure. S5. Concentration-response curves of atrazine on two Ciliophora and two Nauplii copepod larvae. The numbers (1-4) represent the four microzooplankton in the following order: *Oithona similis*, *Paracalanus parvus*, *Euplotes* sp. and *Strombidium* sp.

Figure. S6

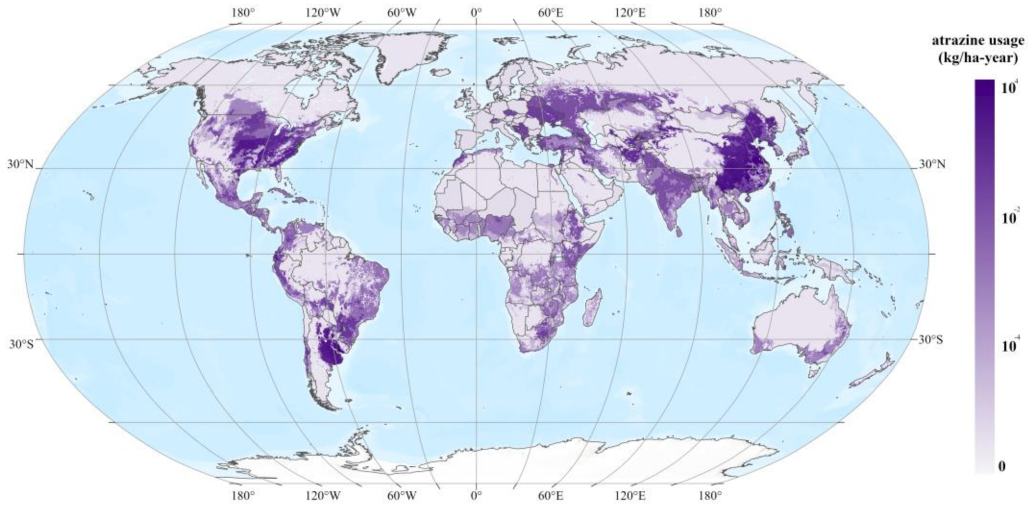

Figure. S6. Global geographic distribution of atrazine usage. The map has a spatial resolution of 5 arcmin, which is approximately 10 km × 10 km at the equator. Atrazine data in each grid cell was based on the usage of atrazine in 175 crops (classified into six dominant crops) in 2015, obtained from CHENGRIDS database. Source data are provided as a Source Data file.

Figure. S7

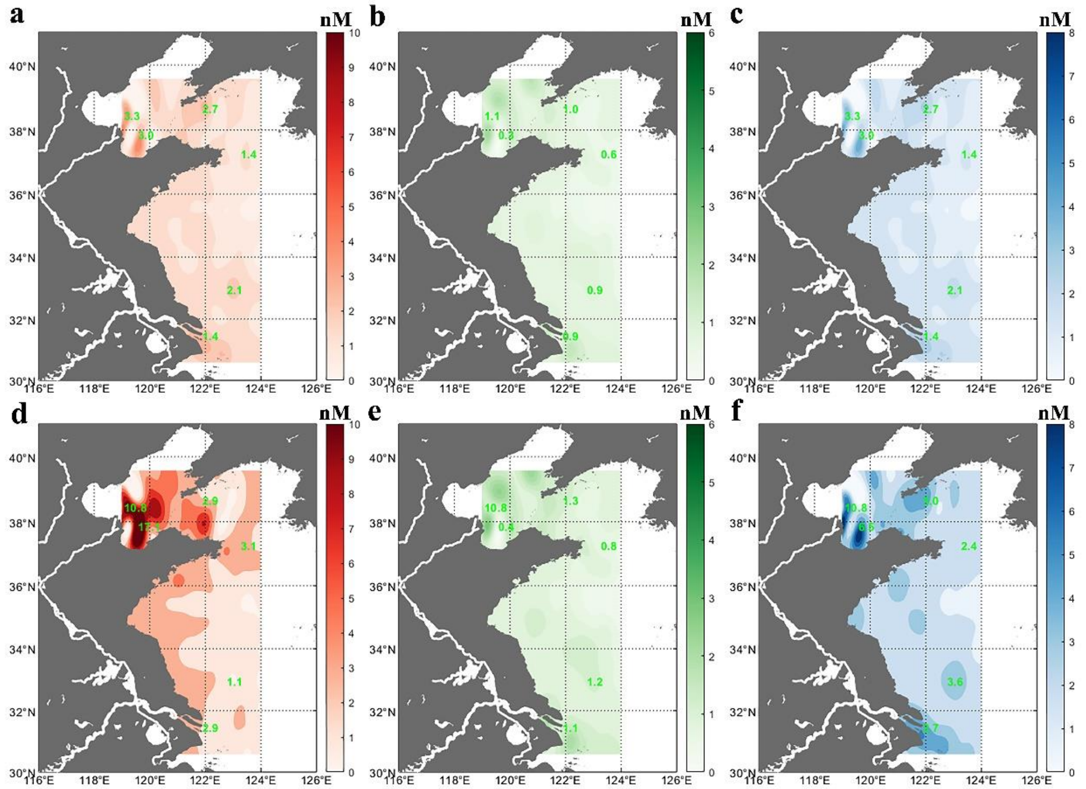

Figure. S7. The distribution of the three typical herbicide classes (triazine, phenylurea, and amide herbicides) in the surface waters of the Bohai Sea and Yellow Sea of China in autumn 2017 (a-c) and spring 2018 (d-f)[25]. (a, d) Triazine herbicides; (b, e) phenylurea herbicides; (c, f) amide herbicides. The colors represent high (red/green/blue) and low (gray) concentrations of the three typical herbicide classes. The unit is nmol L^{-1}.

Figure. S8

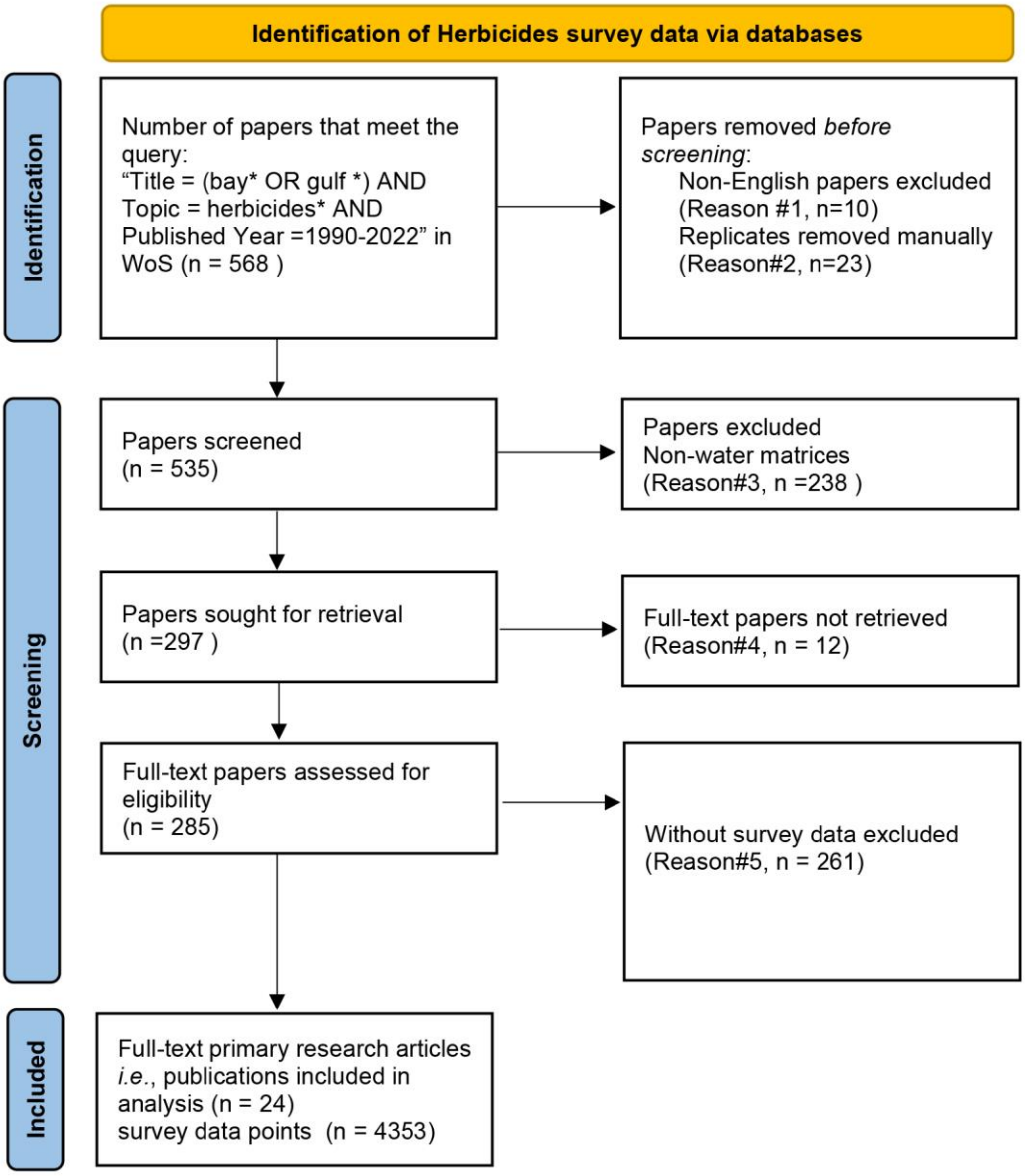

Figure S8. PRISMA flow diagram summarising identification of papers basing on the Web of Science (WoS) Core Collection database.

Figure. S9

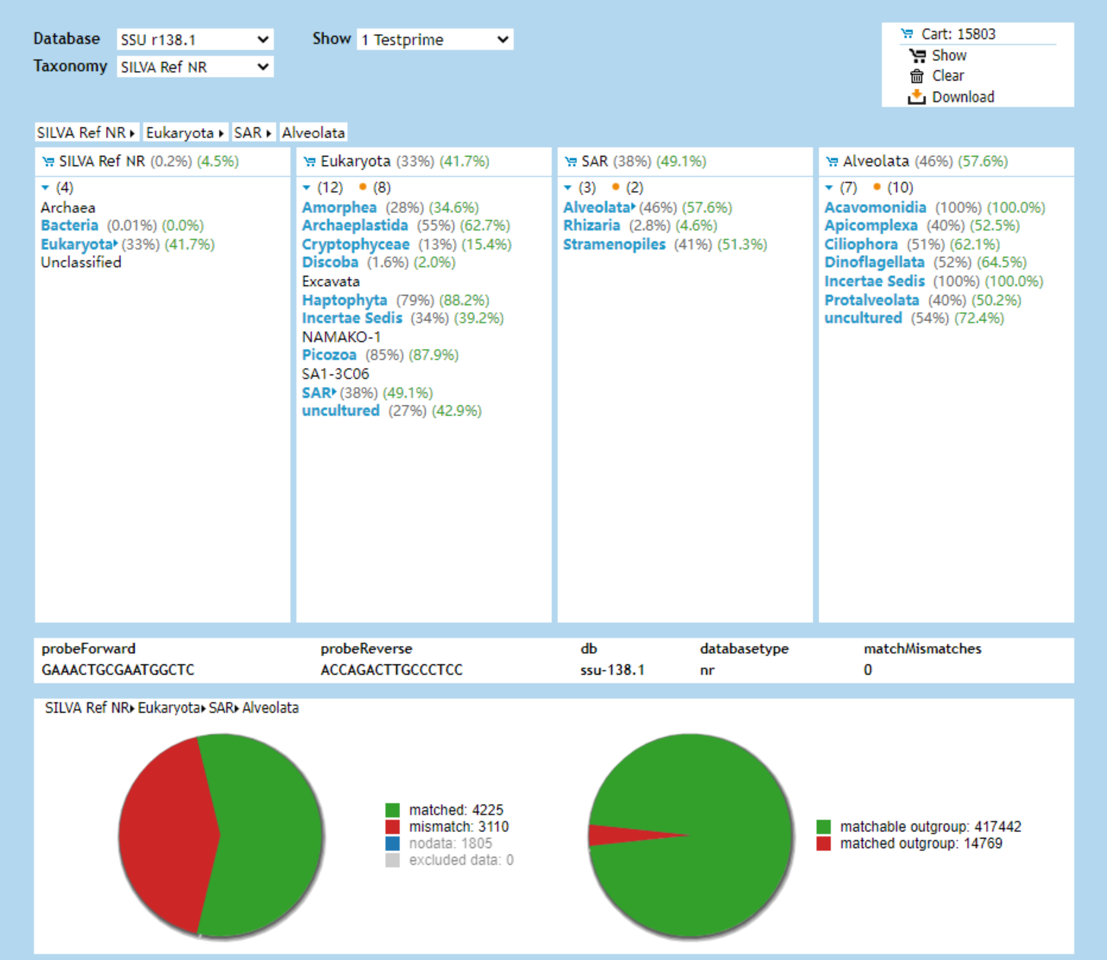

Figure. S9. Screenshot of the Taxonomy Browser showing TestPrime results for the universal primers 82F-516R.

Figure. S10

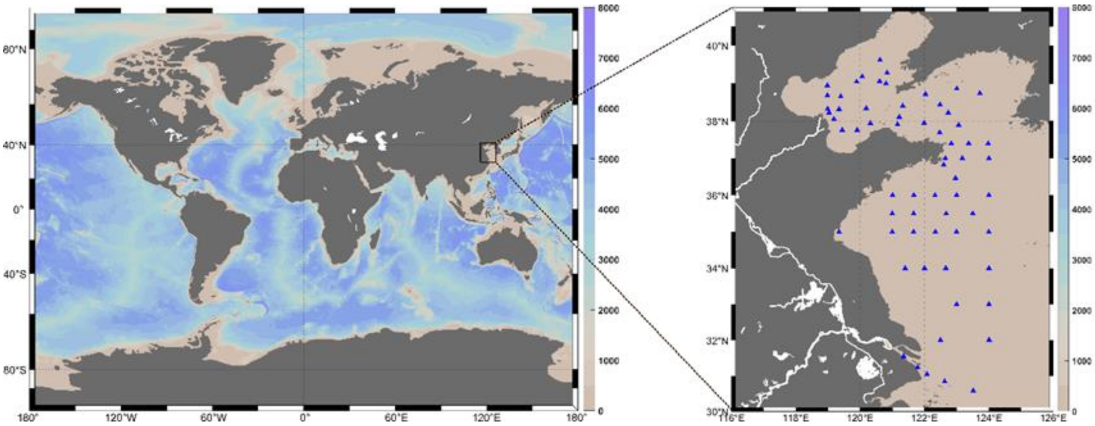

Figure. S10. Map of the study area showing the locations of the 64 sampling sites in the Bohai Sea and Yellow Sea[25]. Source data and the longitude and latitude information of each station are provided as a Source Data file

Reference

1. Jang SH, Jeong HJ, Lee MJ, Kim JH, You JH. Gyrodinium jinhaense n. sp., a new heterotrophic unarmored dinoflagellate from the coastal waters of Korea. *Journal of Eukaryotic Microbiology* **66**, 821-835 (2019).
2. Hoppenrath M, Schweikert M, Elbrächter M. Morphological reinvestigation and characterization of the marine, sand-dwelling dinoflagellate Adenoides eludens (Dinophyceae). *European Journal of Phycology* **38**, 385-394 (2003).
3. Jung JH, Choi JM, Coats DW, Kim YO. Euduboscquella costata n. sp.(Dinoflagellata, Syndinea), an intracellular parasite of the ciliate Schmidingerella arcuata: morphology, molecular phylogeny, life cycle, prevalence, and infection intensity. *Journal of Eukaryotic Microbiology* **63**, 3-15 (2016).
4. Kim E, Archibald JM. Ultrastructure and molecular phylogeny of the cryptomonad Goniomonas avonlea sp. nov. *Protist* **164**, 160-182 (2013).
5. Siano R, Kooistra WH, Montresor M, Zingone A. Unarmoured and thin-walled dinoflagellates from the Gulf of Naples, with the description of Woloszynskia cincta sp. nov.(Dinophyceae, Suessiales). *Phycologia* **48**, 44-65 (2009).
6. Place AR, Bowers HA, Bachvaroff TR, Adolf JE, Deeds JR, Sheng J. Karlodinium veneficum—The little dinoflagellate with a big bite. *Harmful Algae* **14**, 179-195 (2012).
7. Hansen G. Analysis of the thecal plate pattern in the dinoflagellate Heterocapsa rotundata (Lohmann) comb. nov.(= Katodinium rotundatum (Lohmann) Loeblich). *Phycologia* **34**, 166-170 (1995).
8. Eikrem W, Throndsen J. The morphology of Chrysochromulina rotalis sp. nov.(Prymnesiophyceae, Haptophyta), isolated from the Skagerrak. *Sarsia* **84**, 445-449 (1999).
9. Estep K, Davis PG, Hargraves PE, Sieburth J. Chloroplast containing microflagellates in natural populations of north Atlantic nanoplankton, their identification and distribution; including a description of five new species of Chrysochromulina (Prymnesiophyceae). *Protistologica (Paris 1965)* **20**, 613-634 (1984).
10. Rhodes Lesley L, Gyoon KH. The comparison of two strains of Fibrocapsa japonica (Raphidophyceae) in New Zealand and Japan. *Fisheries and Aquatic Sciences* **2**, 58-65 (1999).
11. Takahashi K, Benico G, Lum WM, Iwataki M. Gertia stigmatica gen. et sp. nov.(Kareniaceae, Dinophyceae), a new marine unarmored dinoflagellate possessing the peridinin-type chloroplast with an eyespot. *Protist* **170**, 125680 (2019).
12. Ma Z, Hu Z, Deng Y, Shang L, Gobler CJ, Tang YZ. Laboratory Culture-Based Characterization of the Resting Stage Cells of the Brown-Tide-Causing Pelagophyte, Aureococcus anophagefferens. *Journal of Marine Science and Engineering* **8**, 1027 (2020).
13. Meunier A. *Microplankton de la mer Flamande*. Hayez, imprimeur de l'Académie royale de Belgique (1921).
14. Durbin E. Aspects of the biology of resting spores of Thalassiosira nordenskioeldii and Detonula confervacea. *Marine Biology* **45**, 31-37 (1978).
15. Song W, Wilbert N. Morphological investigations on some free living ciliates (Protozoa, Ciliophora) from China Sea with description of a new hypotrichous genus, Hemigastrostyla nov. gen. *Archiv für Protistenkunde* **148**, 413-444 (1997).
16. Hu X, Song W. Morphology and morphogenesis of Holosticha heterofoissneri nov. spec. from the Yellow Sea, China (Ciliophora, Hypotrichida). *Hydrobiologia* **448**, 171-179 (2001).

17. Blake JA, Woodwick KH. Reproduction and larval development of Pseudopolydora paucibranchiata (Okuda) and Pseudopolydora kempi (Southern)(Polychaeta: Spionidae). *The Biological Bulletin* **149**, 109-127 (1975).
18. Milejkovskij S. On the Morphology and Taxonomy of Polychaetes of the Family Chrysopetalidae E. Ehlers, 1864 (Genera Paleanotus L. Schmarda, 1861, Heteropale, HP Johnson, 1897, and Others). *Zoologicheskii zhurnal* **41**, 648-659 (1961).
19. MOON S-Y, Ohtsuka S, Ueda H, Soh HY. Acartia (Odontacartia) ohtsukai Ueda and Bucklin, 2006 (Copepoda, Calanoida, Acartiidae): first record of its occurrence in Korean waters and habitat segregation from its sibling species A. pacifica Steuer, 1915. *Zootaxa* **1841**, 61–64-61–64 (2008).
20. Ferrari FD, Orsi J. Oithona davisae, new species, and Limnoithona sinensis (Burckhardt, 1912)(Copepoda: Oithonidae) from the Sacramento-San Joaquin Estuary, California. *Journal of Crustacean Biology* **4**, 106-126 (1984).
21. Ross A, Newman WA. A review of the Pyrgoma cancellatum species complex (Cirripedia: Pyrgomatidae). *Journal of Natural History* **36**, 407-421 (2002).
22. Escapa CM*, et al.* The distribution and ecological effects of the introduced Pacific oyster Crassostrea gigas (Thunberg, 1793) in northern Patagonia. (2004).
23. Delgado M, Pérez-Camacho A. Comparative study of gonadal development of Ruditapes philippinarum (Adams and Revé) and Ruditapes decussatus (L.)(Mollusca, Bivalvia): Influence of temperature. (2007).
24. Oya Y, Kimura T, Kajihara H. Description of a new species of Paraplehnia (Polycladida, Stylochoidea) from Japan, with inference on the phylogenetic position of Plehniidae. *ZooKeys* **864**, 1 (2019).
25. Yang L, Mou S, Li H, Zhang Z, Jiao N, Zhang Y. Terrestrial input of herbicides has significant impacts on phytoplankton and bacterioplankton communities in coastal waters. *Limnology and Oceanography* **66**, 4028-4045 (2021).
