# Supplementary material for: Herbicide leakage into seawater impacts primary productivity and zooplankton globally
Source: Nat Commun. 2024 Feb 27;15:1783. doi: 10.1038/s41467-024-46059-4 (PMC10899588; doi:10.1038/s41467-024-46059-4)
Supplement: Supplementary file 3 — Reporting Summary [file 41467_2024_46059_MOESM3_ESM.pdf]

nature portfolio

Corresponding author(s): Liqiang Yang
Last updated by author(s): Jan 30, 2024

Reporting Summary

Nature Portfolio wishes to improve the reproducibility of the work that we publish. This form provides structure for consistency and transparency in reporting. For further information on Nature Portfolio policies, see our Editorial Policies and the Editorial Policy Checklist.

Statistics

For all statistical analyses, confirm that the following items are present in the figure legend, table legend, main text, or Methods section.

n/a	Confirmed	
☐	☒	The exact sample size (*n*) for each experimental group/condition, given as a discrete number and unit of measurement
☐	☒	A statement on whether measurements were taken from distinct samples or whether the same sample was measured repeatedly
☐	☒	The statistical test(s) used AND whether they are one- or two-sided<br>*Only common tests should be described solely by name; describe more complex techniques in the Methods section.*
☐	☒	A description of all covariates tested
☐	☒	A description of any assumptions or corrections, such as tests of normality and adjustment for multiple comparisons
☐	☒	A full description of the statistical parameters including central tendency (e.g. means) or other basic estimates (e.g. regression coefficient) AND variation (e.g. standard deviation) or associated estimates of uncertainty (e.g. confidence intervals)
☐	☒	For null hypothesis testing, the test statistic (e.g. *F*, *t*, *r*) with confidence intervals, effect sizes, degrees of freedom and *P* value noted<br>*Give P values as exact values whenever suitable.*
☒	☐	For Bayesian analysis, information on the choice of priors and Markov chain Monte Carlo settings
☒	☐	For hierarchical and complex designs, identification of the appropriate level for tests and full reporting of outcomes
☒	☐	Estimates of effect sizes (e.g. Cohen's *d*, Pearson's *r*), indicating how they were calculated

Our web collection on statistics for biologists contains articles on many of the points above.

Software and code

Policy information about availability of computer code

Data collection	Based on the Web of Science (WoS) Core Collection database, a total of 568 papers related to herbicides in bays were retrieved with the following search string: "Title = (bay* OR gulf *) AND Topic = herbicides* AND Published Year =1990-2022".<br>The data of global georeferenced, crop-specific, annual herbicide application rates were obtained from the PEST-CHEMGRIDSv1 global database (https://doi.org/10.7927/weq9-pv30).<br>The PCR samples were sent to Shanghai Majorbio Bio-Pharm Technology Co., Ltd. (Shanghai, China) and sequenced (2×300) on an Illumina MiSeq platform.

Data analysis

Raw sequencing data were quality-filtered using QIIME software (v1.8), and the paired reads were merged using FLASH software (v1.2.7). Sequences meeting the following three criteria were included in the downstream analyses: 200< sequence length <600, mean quality >30, ambiguous bases <1, and homopolymer length <6. Erroneous and chimeric sequences were further eliminated by USEARCH. Then, the remaining sequences were clustered into operational taxonomic units (OTUs) according to a 97% sequence similarity threshold using UCLUST (v1.2.22). OTUs containing fewer than five sequences were eliminated. The same number of sequences (30,000) were randomly selected from each sample for standardization.

When the herbicides data in the papers were presented in graphical form, Plot Digitizer 3.3 software was used to extract the data from the figures.

The phytoplankton and micro-zooplankton OTUs that were significantly ($p<0.05$) affected by at least one atrazine level (0.5, 5, or 50 nmol L-1) were used in co-occurrence network analysis. The relative abundances of phytoplankton and micro-zooplankton OTUs in the control and atrazine-treated groups were employed to construct Bray-Curtis similarity matrices. The Spearman rank correlation coefficients between the phytoplankton and micro-zooplankton taxa were calculated in R (version 3.5.3). To reduce noise and the occurrence of false-positive predictions, only strong correlations ($|r| \geq 0.9$, $p \leq 0.05$) were selected for Gephi network visualization (version 0.9.2).

For manuscripts utilizing custom algorithms or software that are central to the research but not yet described in published literature, software must be made available to editors and reviewers. We strongly encourage code deposition in a community repository (e.g. GitHub). See the Nature Portfolio guidelines for submitting code & software for further information.

Data

Policy information about availability of data

All manuscripts must include a data availability statement. This statement should provide the following information, where applicable:

- Accession codes, unique identifiers, or web links for publicly available datasets
- A description of any restrictions on data availability
- For clinical datasets or third party data, please ensure that the statement adheres to our policy

The raw sequencing data generated in this study have been deposited in the National Center for Biotechnology Information (NCBI) Sequence Read Archive (SRA, http://www.ncbi.nlm.nih.gov/Traces/sra/sra.cgi) under accession number PRJNA913270 (https://dataview.ncbi.nlm.nih.gov/object/PRJNA913270). The data of global georeferenced, crop-specific, annual herbicide application rates were obtained from the PEST-CHEMGRIDSv1 global database (https://doi.org/10.7927/weq9-pv30). Herbicide data are available in Supplementary Tables 1, 2 and 5. Source data are provided with this paper.

Research involving human participants, their data, or biological material

Policy information about studies with human participants or human data. See also policy information about sex, gender (identity/presentation), and sexual orientation and race, ethnicity and racism.

Reporting on sex and gender	n/a
Reporting on race, ethnicity, or other socially relevant groupings	n/a
Population characteristics	n/a
Recruitment	n/a
Ethics oversight	n/a

Note that full information on the approval of the study protocol must also be provided in the manuscript.

Field-specific reporting

Please select the one below that is the best fit for your research. If you are not sure, read the appropriate sections before making your selection.

☐ Life sciences ☐ Behavioural & social sciences ☒ Ecological, evolutionary & environmental sciences

For a reference copy of the document with all sections, see nature.com/documents/nr-reporting-summary-flat.pdf

Ecological, evolutionary & environmental sciences study design

All studies must disclose on these points even when the disclosure is negative.

Study description	This study aims to reveal the current global status of marine herbicide pollution and evaluate its impacts on marine primary productivity and secondary effects on higher trophic levels. By analyzing the spatiotemporal distribution of herbicides at 661 gulf stations worldwide from 1990 to 2022, an overall picture of the current status of herbicide pollution in global coastal waters was obtained; by establishing the toxicity equivalent database of each herbicide and the dose-response relationship between the concentration of atrazine and chlorophyll a in seawater at the phytoplankton community level, the overall inhibition effect of 12 triazine herbicides on phytoplankton primary productivity was quantified; by analyzing the effects of herbicides on phytoplankton community structure, particle size composition, production cycle, and energy transfer process, the potential mechanism of herbicides inhibiting phytoplankton primary productivity was elucidated; Moreover, the effect of herbicides on higher trophic levels and the possibility of predicting marine herbicide pollution through indicators of herbicide use on land were explored.
Research sample	Herbicide residues: To quantify the impact of current herbicide pollution on offshore primary productivity on a larger scale, we first collected survey data published from 1995 to 2022 on herbicide pollution in bays around the world. The temporal and spatial distribution patterns and background values of herbicides in the coastal waters of typical bay areas on all continents were determined.<br><br>Phytoplankton community: exploring the effects of the suppression of sensitive algae on the abundance, community composition, and particle size of phytoplankton is expected to explain the effects of modern intensive agriculture on marine primary production.<br><br>Application rates (ARs) of herbicides: The data of global georeferenced, crop-specific, annual herbicide application rates were obtained from the PEST-CHEMGRIDSv1 global database (https://doi.org/10.7927/weq9-pv30) to draw the geographic distribution map and ecological risk level map of global herbicide usage.
Sampling strategy	Data collection of the spatiotemporal distribution of herbicide residues in typical bays worldwide: a total of 568 papers related to herbicides in bays were retrieved with the following search string: "Title = (bay* OR gulf *) AND Topic = herbicides* AND Published Year =1990-2022". Data on the types and concentrations of herbicides in each bay were collected in each article, as well as the survey dates and geographical location. When the data in the papers were presented in graphical form, Plot Digitizer 3.3 software was used to extract the data from the figures.<br>Phytoplankton community collection: On 5 July 2018, 1200 liters of clean seawater (with no detected herbicide residue) was collected from the estuary of Shilaoren Bay (120°49′E, 36°09′N), Qingdao, China, and filtered through a 200 µm-mesh net to remove large particles. In the laboratory, four atrazine doses (0, 0.5, 5 and 50 nmol L-1) were used in the treatment groups: 1) control check (CK), consisting of uncontaminated seawater; 2) 0.5 nmol L-1 atrazine; 3) 5 nmol L-1 atrazine; and 4) 50 nmol L-1 atrazine. Each treatment sample consisted of 80 L of seawater in a transparent polycarbonate bottle (100 L), and each treatment had three replicates (Supplementary Fig. S8). The mouth of each bottle was covered with a parafilm membrane to ensure gas exchange while preventing contamination. The bottles were incubated at room temperature (25°C±3°C) for 30d in the laboratory under natural light conditions, and subsamples were collected from each bottle on days 0, 1, 2, 4, 7, 14, 21 and 30 for subsequent analyses.<br>Analysis of herbicide residues in waters: 400mL of water treatment was adjusted to pH 7 and filtered through a 0.7-µm GF/F filter (47mm). Then, 10 µL of internal standard solution (acetonitrile) containing 1 µmolL−1 atrazine D5, diuron D6, and metolachlor D6 was added. After successive activation with methanol (10 mL) and deionized water (10 mL), Oasis HLB cartridges (Milford) were employed to preconcentrate the analytes from the water samples, with a vacuum maintained at 400mmHg with a Vac Elut SPS 24 vacuum manifold (Agilent Technology).
Data collection	Data collection of the spatiotemporal distribution of herbicide residues in typical bays worldwide: X.H. collected the data of the spatiotemporal distribution of herbicide residues in typical bays worldwide. a total of 568 papers related to herbicides in bays were retrieved with the following search string: "Title = (bay* OR gulf *) AND Topic = herbicides* AND Published Year =1990-2022". Data on the types and concentrations of herbicides in each bay were collected in each article, as well as the survey dates and geographical location. When the data in the papers were presented in graphical form, Plot Digitizer 3.3 software was used to extract the data from the figures.<br><br>The V1-V3 hypervariable region of the phytoplankton 18S rRNA gene was amplified with the eukaryote-specific primers 18S-82F (5′-GAAACTGCGAATGGCTC-3′) and Ek-516R (5′-ACCAGACTTGCCCTCC-3′) 83. The coverage and specificity of this primer set for the major taxonomic groups of phytoplankton and micro-zooplankton were evaluated using SILVA TestPrime 1.0 with version 138.1 of the SILVA SSU Ref database 84, with no mismatches allowed. This primer set was previously shown to theoretically amplify the 18S rDNA region from all major taxonomic groups (Supplementary Fig. S9). The PCR samples were sent to Shanghai Majorbio Bio-Pharm Technology Co., Ltd. (Shanghai, China) and sequenced (2×300) on an Illumina MiSeq platform. The DNA concentrations in parallel incubations of blank filters were below the detection limit, and no detectable amplification of 18S rRNA gene products was observed.<br><br>L.Y. retrieved the average usage data of 52 herbicides that are currently being widely used worldwide from the PEST-CHEMGRIDSv1 global database (https://doi.org/10.7927/weq9-pv30), and reanalyzed them with matlab software (MathWorks, Inc., USA) to draw the geographic distribution map and ecological risk level map of global herbicide usage.
Timing and spatial scale	The collection period of herbicide residue data in coastal waters is from June 1990 to December 2022, covering 15 typical bays around the world. The 15 bays were clustered into seven sea areas, including the east coast of United States, the Gulf of Mexico, France, the Mediterranean Sea, South Africa, East Asia and Australia.<br>On 5 July 2018, seawater (with no detected herbicide residue) was collected from the estuary of Shilaoren Bay (120°49′E, 36°09′N), Qingdao, China.<br>The four experimental micro-zooplankton were isolated from the coastal waters of Shilaoren Bay (120°49′E, 36°09′N) and cultured in artificial seawater medium on 17 July 2021.

	The top 20 herbicides used for each crop class in 2015 were selected as the statistical range of the global herbicide geographical distribution.
Data exclusions	Data on non-seawater substrates were excluded
Reproducibility	The author's laboratory carried out a large amount of relevant research work before and after obtaining the experimental results described in this article. The results show that the reproducibility of the microscopic experimental results involved in this experiment is good.
Randomization	The experimental samples (seawater, phytoplankton, etc.) involved in this experiment were thoroughly mixed and then randomly allocated to the atrazine-treated group or control group. All culture bottles were randomly placed in the incubator. for each culture, samples were taken after the culture was gently and thoroughly mixed to make sure they represent the entire culture.
Blinding	Samples for measurements and analyses were blinded so that no information about the culture condition was indicated on the samples during the measurements and analyses.

Did the study involve field work? ☐ Yes ☒ No

Reporting for specific materials, systems and methods

We require information from authors about some types of materials, experimental systems and methods used in many studies. Here, indicate whether each material, system or method listed is relevant to your study. If you are not sure if a list item applies to your research, read the appropriate section before selecting a response.

Materials & experimental systems

n/a	Involved in the study
☒	☐ Antibodies
☒	☐ Eukaryotic cell lines
☒	☐ Palaeontology and archaeology
☒	☐ Animals and other organisms
☒	☐ Clinical data
☒	☐ Dual use research of concern
☒	☐ Plants

Methods

n/a	Involved in the study
☒	☐ ChIP-seq
☒	☐ Flow cytometry
☒	☐ MRI-based neuroimaging
